# In Silico Approach Triterpene Glycoside of *H. atra* Targeting Orotidine 5-Monophosphate Decarboxylase Protein (PfOMPDC) in *P. falciparum* Infection Mechanism

**DOI:** 10.1155/2024/5924799

**Published:** 2024-04-01

**Authors:** Prawesty Diah Utami, Herin Setianingsih, Dewi Ratih Tirto Sari

**Affiliations:** ^1^Parasitology Departement, Faculty of Medicine, Hang Tuah University, Surabaya, Indonesia; ^2^Anatomy and Histology Departement, Faculty of Medicine, Hang Tuah University, Surabaya, Indonesia; ^3^Pharmacy Department, Faculty of Medical Science, Ibrahimy University, Situbondo, Indonesia

## Abstract

This study accessed the potential antimalarial activity of triterpene glycoside of *H. atra* through targeting orotidine 5-monophosphate decarboxylase protein (PfOMPDC) in *P. falciparum* by molecular docking. Nine triterpene glycosides from *H. atra* extract modeled the structure by the Corina web server and interacted with PfOMPDC protein by using Hex 8.0.0. The docking results were visualized and analyzed by Discovery Studio version 21.1.1. 17-Hydroxyfuscocineroside B showed the lowest binding energy in PfOMPDC interaction, which was -1,098.13 kJ/mol. Holothurin A3, echinoside A, and fuscocineroside C showed low binding energy. Nine triterpene glycosides of *H. atra* performed interaction with PfOMPDC protein at the same region. Holothurin A1 posed interaction with PfOMPDC protein by 8 hydrogen bonds, 3 hydrophobic interactions, and 8 unfavorable bonds. Several residues were detected in the same active sites of other triterpene glycosides. Residue TYR111 was identified in all triterpene glycoside complexes, except holothurin A3 and calcigeroside B. In summary, the triterpene glycoside of *H. atra is* potentially a drug candidate for malaria therapeutic agents. In vitro and in vivo studies were required for further investigation.

## 1. Introduction

Malaria remains a persistent health issue, resulting in significantly elevated rates of illness and mortality, as evidenced by consecutive publications of the World Malaria Report. Based on the most recent research, the global incidence of cases in 2021 was anticipated to be approximately 247 million, with a corresponding mortality rate of 619,000 deaths [1, 2]. The primary objective on a worldwide scale is to decrease the impact of this disease on public health and mortality rates, while also maintaining the ultimate goal of eradicating malaria in the long run [3]. Malaria is a potentially fatal illness resulting from the invasion of erythrocytes by hemoprotozoan parasites belonging to the *Plasmodium* genus. These parasites are transferred to humans by the bites of female Anopheles mosquitoes that are infected. The human population is most usually infected by four distinct species of *Plasmodium*. In addition to *P. ovale* and *P. malariae*, *P. falciparum*, and *P. vivax* are usually acknowledged as the most widespread species, with *P. falciparum* being particularly notorious for its high pathogenicity [4]. The presence of a fifth species, known as *P. knowlesi*, has been observed in human populations in Southeast Asia and the Western Pacific regions, with a notable concentration on the island of Borneo. *P. knowlesi* is a species of parasite that typically infects primate species other than humans [5].

Certain individuals exhibit a higher susceptibility to the development of severe malaria in comparison to others. Infants and toddlers below the age of five, pregnant women, and individuals diagnosed with HIV/AIDS are particularly vulnerable to the associated risks. Additional vulnerable populations consist of individuals who are entering regions with high levels of malaria transmission but have not developed partial immunity due to prolonged exposure to the disease. This may include migrants, mobile communities, and travelers who are not utilizing chemopreventive medicines [3, 6]. The World Health Organization (WHO) advocates for the timely identification of those displaying symptoms indicative of malaria. Failure to administer prompt treatment for *P. falciparum* malaria within a 24-hour timeframe may result in the infection's progression to severe morbidity and ultimately mortality [3]. Severe malaria has been observed to induce multiorgan failure in adults, whereas children commonly experience severe anemia, respiratory distress, or cerebral malaria. Infection with several *Plasmodium* species other than *P. falciparum* can lead to substantial morbidity and, in some cases, pose a risk to human life [7].

Artemisinin-based combination treatments (ACTs) represent the current pinnacle of efficacy among antimalarial medications and are considered the primary treatment modality for *P. falciparum* malaria, which is recognized as the most lethal strain of malaria worldwide [8]. In the absence of any foreseeable alternatives to artemisinin derivatives in the near future, it is imperative to safeguard the effectiveness of ACTs. In recent years, the issue of parasite resistance to antimalarial drugs has become a significant concern in the battle against malaria, with a particular focus on the Greater Mekong subregion [9]. WHO has expressed its concern about the emerging evidence of drug-resistant malaria in Africa. Thus far, evidence has been gathered indicating the presence of resistance in three of the five malaria species identified: *P. falciparum*, *P. vivax*, and *P. malariae* [3]. The term “partial resistance to artemisinin” often denotes a prolongation in eliminating malaria parasites from the circulatory system after the administration of ACTs. Consequently, the efficacy of the artemisinin compound in eradicating all parasites within a 72-hour timeframe is diminished in individuals afflicted with malaria strains that exhibit partial resistance to artemisinin [10].

The emergence of ACT resistance has resulted in therapeutic failures and a disruption of the transmission cycle, leading to an escalation in both the morbidity and fatality rates associated with malaria. Scientists do research to identify therapeutic compounds derived from natural sources. It has been scientifically demonstrated that marine organisms possess a diverse range of bioactive compounds that confer health benefits. The species known as *Holothuria atra* is a sea cucumber characterized by its reddish-black coloration. Its dorsal surface is adorned with numerous small and closely packed long papillae [11]. The sea cucumbers under consideration are distributed across the shallow waters of Indonesia, with a particular affinity for regions close to sandy shores and coral reefs. It is known that *H. atra* contains a variety of active compounds, some of which are chlorogenic acid, coumaric acid, ascorbic acid, rutin, pyrogallol, artemisinin, and catechin [12]. *H. atra* has been included in the community's dietary practices and has been found to possess several bioactive compounds that exhibit antifungal and antibacterial effects [13–15].

In a previous study, the efficacy of *H. atra* as an antimalarial agent was examined by an in silico approach targeting the PfOMPDC/orotidine 5-monophosphate decarboxylase protein of the *P. falciparum* [16]. Based on other research, using the in vitro method revealed that *H. atra* extract has high antimalarial activity, with an IC_50_ value of 1.23 *μ*g/mL indicating its significant efficacy in inhibiting parasite development [8]. Based on the observed phenomenon, it is anticipated that *H. atra* possesses the potential to serve as an alternative therapeutic agent for combating malaria. In order to ascertain the efficacy of *H. atra* as an antimalaria medication, this research employs the in silico approach to discern the capacity of the triterpene glycoside of *H. atra* to impede the essential protein activity of *P. falciparum*, hence hindering the proliferation and maturation of this parasite.

The field of in silico pharmacology is expanding quickly around the world. It is the study of how to use software to collect, analyze, and combine biological and medical data from a wide range of sources. To be more precise, it establishes the use of this data in developing computational models or simulations that can be employed to create forecasts, propose hypotheses, and ultimately yield breakthroughs or advancements in the field of medicine and therapies [17]. The utilization of the silico method has emerged as a rapid and cost-effective research approach for the exploration of potential remedies for novel diseases such as COVID-19 [18, 19]. This research employed an in silico approach to forecast the efficacy of triterpene glycoside of *H. atra* in suppressing the *P. falciparum* protein, PfOMPDC.

## 2. Experimental

### 2.1. Compound Retrieving and Modeling

Triterpene glycoside compounds were isolated from *H. atra* and retrieved their structures from the PubChem and NCBI databases (Table 1). The 3D structure model was carried out from the Corina web server (https://demos.mn-am.com/corina.html). The structures of compounds were downloaded in Protein Data Bank file format and used for docking.

### 2.2. Protein Retrieval and Preparation

The protein under investigation is the orotidine 5-monophosphate decarboxylase protein derived from the *Plasmodium falciparum* parasite, referred to as PfOMPDC which downloaded the structure from the Protein Data Bank in pdb file format. The accession code of the protein was 2ZA1 (https://www.rcsb.org/structure/2ZA1) [17]. The protein structure was imported to the Discovery Studio version 21.1.1, then removed the water, hetatm, and ligand from protein [18–26].

### 2.3. Pharmacokinetics, Toxicity, and Druglikeness Prediction

Forecasting of pharmacokinetics (absorption, distribution, metabolism, and excretion process), level of toxicity, and druglikeness characteristics is a key feature in computer-aided drug discovery for screening compounds [27, 28]. Targeted ligands, triterpene glycoside compounds of *H. atra*, predicted the pharmacokinetic properties using the SwissADME online program [27, 29–31]. The compounds also identified the toxicity by using the ProToxII program (https://tox-new.charite.de/protox_II/index.php?site=home) [28, 32, 33], and also, genetic mutation, cancer-causing capacity, skin sensitivity, and other toxicological features had been predicted [34, 35]. Druglikeness was predicted to identify the potential drug candidates of triterpene glycosides of *H. atra*. The druglikeness prediction was carried out by SwissADME [27, 30].

### 2.4. Molecular Docking and Visualization

Nine triterpene glycoside compounds from *H. atra* interacted with PfOMPDC protein. Molecular docking was carried out by Hex Cuda 8.0.0 [34]. The docking control was configured with correlation kinds including shape, electro, and DARS. The compute device's CPU was utilized, with 3D as the Fast Fourier Transform (FFT) mode. The sampling angle was set to cover a variety of angles. The postprocessing parameter was configured as none, with a grid dimension of 0.6. The solution count was set to 2,000, while the receptor and ligand ranges were both set to 180. The step size for both ligand and receptor was set to 7.5 [36]. The twist range was set to 360 with a step size of 5.5. The distance range was set to 40, and the box size was set to 10. The translation step was set to 0.8, with no substeps. The score threshold was set to 0, and a steric scan was performed with a range of 18, soln 1, and final search 25. Docking results were viewed by Discovery Studio version 21.1.1. The analyzed data were 3D complex structure, hydrophobicity, hydrogen bond profile, and interaction of ligand–protein structure.

### 2.5. Molecular Dynamic

The lowest total binding energy of ligand–protein complex was selected for molecular dynamics. Orotidine-5′-monophosphate was used as a control. The dynamic was carried out by SiBioLEAD tools (https://sibiolead.com/#about) with preprocessing parameter forcefield OPLS/AA, water simple point charge, and box type triclinic, neutralized by NaCl with 0.15 mM. Energy minimization sets the EM integrator steepest descent with the number of EM steps that was 5,000 [35]. Equilibration type NVT/NPT includes temperature 300 K, pressure 1.0 bar, 100 ps. The simulation parameter that was used in this study was integrator leap frog with 1 ns simulation time and 5,000 saved frame numbers. The recorded measurements in the molecular dynamics study consisted of the root mean square deviation (RMSD) and the radius of gyration, binding free energy estimation (MMPBSA), and hydrogen bonds of protein–ligand.

## 3. Results

Pharmacokinetic properties, LD50, and toxicity class of triterpene glycoside of *H. atra* compounds were presented in Table 1. 17-Hydroxyfuscocineroside B and holothurin A3 have high LD50 with 800 mg/kg, classified as toxicity class 4, while the other compounds were classified as toxicity class 5. As the potency or toxicity of a substance increases, the LD50 decreases, and the dose required to induce mortality decreases. Seven bioactive components, with the exception of 17-hydroxyfuscocineroside B and holothurin A3, possess toxicity class 5 properties which indicate minimal toxicities. The findings of this investigation align with the results of a prior toxicity test on *H. atra* conducted by Moelyadi et al. [16], in which it was observed that five active constituents of *H. atra* exhibited toxic properties of a five on the same scale as the control drug, artemisinin, which serves as the standard for malaria. All triterpene glycoside compounds of *H. atra* performed low gastrointestinal absorption, disabled blood-brain barrier permeability, and inactive CYP1A2, CYP2D6, and CYP3A4 inhibitors that do not interfere with cytochrome P450's work. Skin permeation of triterpene glycoside compounds was varied. 17-Hydroxyfuscocineroside B, holothurin A1, calcigeroside B, fuscocineroside C, and echinoside A showed similar value of LogKP. Interstingly, holothurin A3, as a derivative compound of holothurin A, showed lower LogKP.

**Figure 1:**
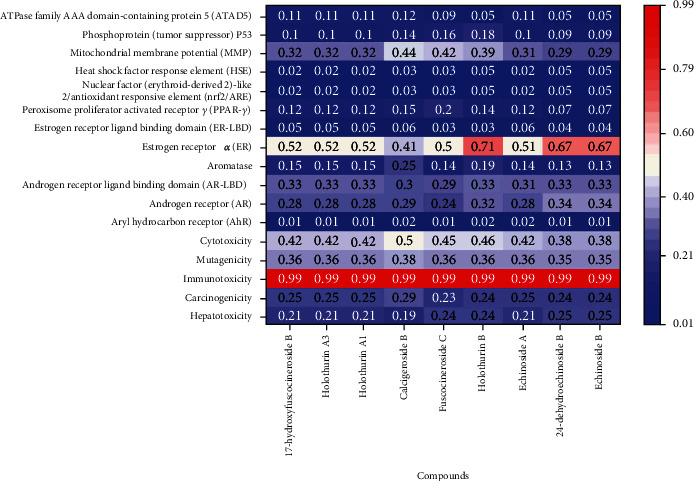
Toxicity performance of triterpene glycoside compounds of *H. atra*.

The toxicity effect of triterpene glycoside compounds performed high immunotoxicity with a potential toxicity of 0.99 (Figure 1). 17-Hydroxyfuscocineroside B, holothurin A3, holothurin A1, fuscocineroside C, holothurin B, echinoside A, 24-dehydroechinoside B, and echinoside B have potential toxicity as estrogen receptor alpha (ER) with value more than 0.5. Cytotoxicity, mutagenicity, carcinogenicity, and hepatotoxicity of triterpene glycoside were lower than 0.4, indicating inactive toxicity.

Table 2 represents the druglikeness, bioavailability, and binding energy of nine triterpene glycoside compounds against *P. falciparum* orotidine 5-monophosphate decarboxylase (PfOMPDC) protein. Based on the druglikeness, nine triterpene glycoside compounds were not potentially as drugs due to Lipinski, Ghose, Veber, Egan, and Muegge's prediction. The bioavailability also performed low bioavailability with a value less than 0.2.

Molecular docking of triterpene glycoside toward orotidine 5-monophosphate decarboxylase protein of the *P. falciparum* (PfOMPDC) protein performed interaction, hydrophobicity, hydrogen bonds, and binding energy. The binding energy of binding affinity was shown in kJ/mol. 17-Hydroxyfuscocineroside B showed the lowest binding energy in PfOMPDC interaction, which was -1,098.13 kJ/mol. Holothurin A3, echinoside A, and fuscocineroside C showed low binding energy (Table 2). Interestingly, holothurin A3, holothurin A1, and holothurin B, even similar structure and derivative compounds, performed different binding energies when interacting with PfOMPDC. Similar results with echinoside A and B, echinoside B had two times higher binding energy than echinoside A.

**Figure 2:**
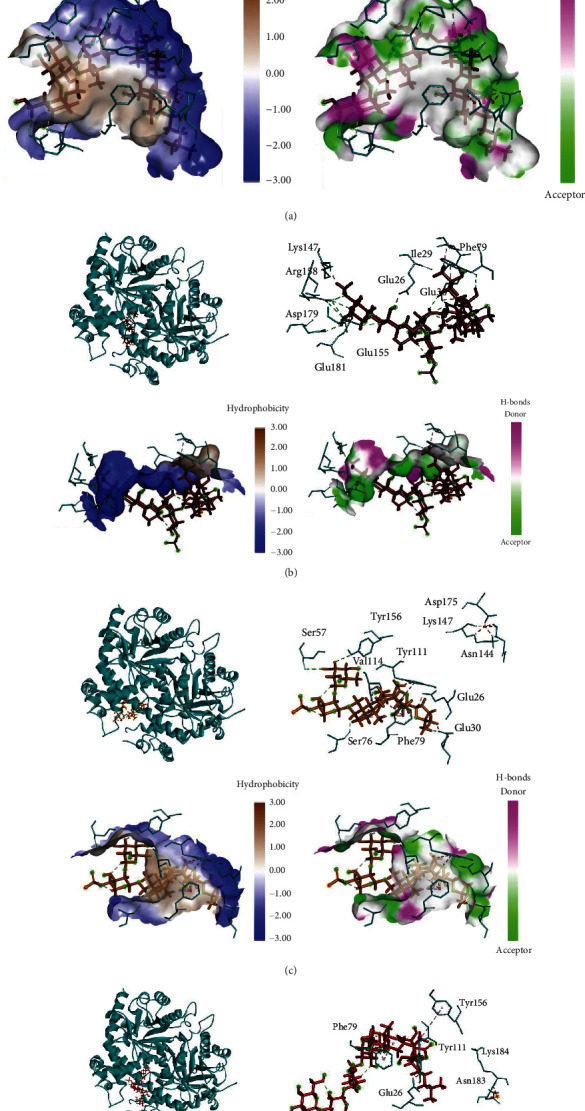
(a) The 3D complex structure, hydrophobic profile, and hydrogen bonds of holothurin A1–protein interaction. (b) The 3D complex structure, hydrophobic profile, and hydrogen bonds of holothurin A3–protein interaction. (c) The 3D complex structure, hydrophobic profile, and hydrogen bonds of holothurin B–protein interaction. (d) The 3D complex structure, hydrophobic profile, and hydrogen bonds of echinoside A–protein interaction.

**Figure 3:**
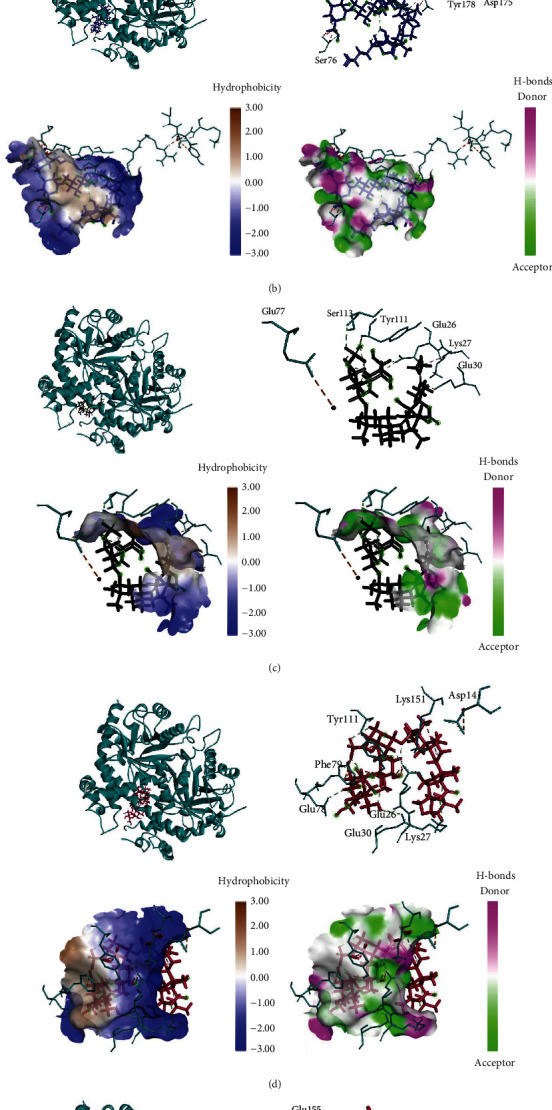
(a) The 3D complex structure, hydrophobic profile, and hydrogen bonds of echinoside B–protein interaction. (b) The 3D complex structure, hydrophobic profile, and hydrogen bonds of 17-hydroxyfuscocineroside B–protein interaction. (c) The 3D complex structure, hydrophobic profile, and hydrogen bonds of 24-dehydroechinoside B–protein interaction. (d) The 3D complex structure, hydrophobic profile, and hydrogen bonds of fuscocineroside C–protein interaction. (e) The 3D complex structure, hydrophobic profile, and hydrogen bonds of calcigeroside B–protein interaction.

Nine triterpene glycosides of *H. atra* performed interaction with PfOMPDC protein at the same region (Figures 2 and 3). Holothurin A1 posed interaction with PfOMPDC protein by 8 hydrogen bonds, 3 hydrophobic interactions, and 8 unfavorable bonds. Several residues were detected in the same active sites of other triterpene glycosides. Residue TYR111 was identified in all triterpene glycoside complexes, except holothurin A3 and calcigeroside B (Figure 3(e)). TYR156 was also showed at the active site of holothurin B (Figure 2(c)). GLU77 was also identified at hydrogen bonds of holothurin A1 and electrostatic interaction of 24-dehydroechinoside B (Figure 3(c)). The hydrophobicity and the hydrogen bond profiles of all ligand–protein interactions showed similarities as shown in Figures 2 and 3.

Holothurin A3, as a derivate compound of holothurin A1, revealed different binding sites of PfOMPDC protein.  Table 3 performs active sites and interaction type of nine terpenoids against PfOMPDC. Holothurin A3 formed a complex with protein by 11 hydrogen bonds, 2 hydrophobic interactions, and 4 unfavorable bonds. Holothurin A3 posed interaction with ARG150 by four hydrogen bonds at the distance 2.6, 2.3, 2.38, and 2.7A. GLU30 was showed at holothurin A1, holothurin A3, and holothurin B. ASP179 and ARG150 were only detected at the holothurin A3 (Figures 2(a)–2(c)). Even though the holothurin A1 and B were similar structures, they were not binding sites. The hydrophobic interaction of holothurin A3 was also found in two residues, which were ILE29 and PHE79. Those two residues were also found at holothurin A1, while only Phe79 was at the same site of holothurin B. Holothurin B revealed some interaction with PfOMPDC protein. The active sites were SER57 (2.57), TYR156 (3.04), SER76 (2.12), and GLU26 (1.83) as hydrogen bond residues; VAL114 (4.4), PHE79 (5.0; 4.6; 4.07), and TYR111 (4.05; 2.24) as hydrophobic interactions; ASN144 (2.2; 1.4), LYS147 (2.1; 2.0; 2.6), TYR156 (0.89), and GLU30 (1.57; 1.55) as unfavorable bonds; and ASP175 (3.06) as electrostatic residue.

Similar to holothurin, echinoside A and echinoside B also showed different interactions with protein. Both echinoside A and B did not show hydrogen bonds (Figure 3). The interaction was hydrophobic interaction, unfavorable, and electrostatic. Echinoside A performed interaction with the binding sites TYR156 (5.4), PHE79 (4.4; 4.5), and TYR111 (4.6) with hydrophobic interaction (Figure 2(d)). Several unfavorable bonds was also showed in residue ASN183 (1.7; 1.6; 2.1; 1.9), LYS184 (2.6), GLU26 (1.6; 1.5), PHE79 (1.8; 1.9), and TYR111 (2.1; 1.8; 1.6; 1.2; 1.3). Echinoside B performed unfavorable bonds at GLU26 (2.2; 1.2; 2.0; 1.1), TYR111 (2.0), and electrostatic at ASP61 (3.5) (Figure 3(a)).

**Figure 4:**
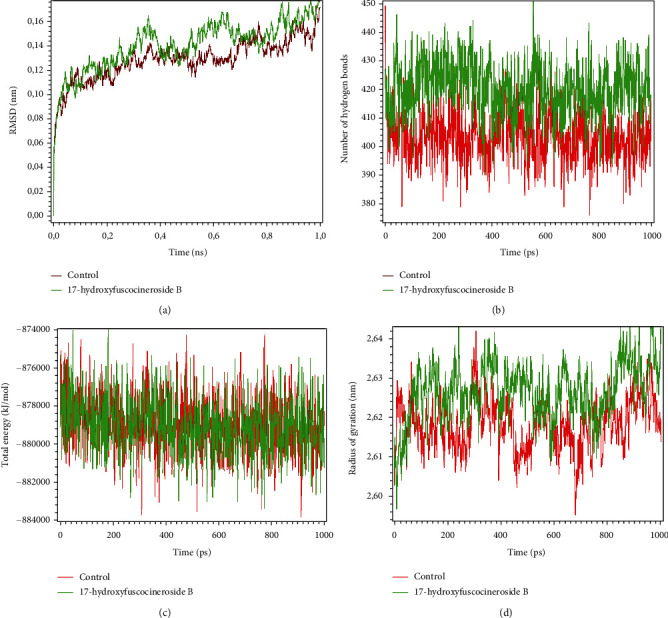
(a) The RMSD of 17-hydroxyfuscocineroside B in comparison with orotidine-5′-monophosphate as a control. (b) The number of hydrogen bonds of 17-hydroxyfuscocineroside B in comparison with orotidine-5′-monophosphate as a control. (c) The total energy of 17-hydroxyfuscocineroside B in comparison with orotidine-5′-monophosphate as a control. (d) The radius of gyration of 17-hydroxyfuscocineroside B in comparison with orotidine-5′-monophosphate as a control.

The 17-hydroxyfuscocineroside B and orotidine-5′-monophosphate were subjected to molecular dynamic simulation. A box of water with NaCl 0.15 mM was used to prepare protein–ligand complex for simulation. The stability of the complex was assessed by simulating 100 ps and analyzed by RMSD, number of hydrogen bonds, radius of gyration, and total binding energy plots. RMSD plots showed that 17-hydroxyfuscocineroside B has multiple binding orientations and increased after 0.2 ns. The RMSD of the complex was lower than 0.2 nm, indicating lower conformational change and high stability of the structure (Figure 4). The number of hydrogen bonds and radius of gyration of 17-hydroxyfuscocineroside B was higher than the control, while the total binding energy both of the compound and control was overlapping.

## 4. Discussion

The malaria parasites depended on nucleotide synthesis. The orotidine 5-monophosphate decarboxylase protein (PfOMPDC) derived from *P. falciparum* played a significant role in the process of nucleotide de novo synthesis, particularly within the context of *P. falciparum*. The two-step requirement for uridine monophosphate synthesis was addiction ribose-5-phosphate to form orotic acid. Orotic acid was converted into orotidine 5-monophosphate which was catalyzed by orotate phosphoribosyltransferase. Then, the orotic acid was decarboxylated by OMP decarboxylase to make uridine 5-monophosphate. The inhibiting de novo synthesis of uridine in *P. falciparum* was an alternative strategy for preventing malaria infection in humans [17, 37–40]. This study performed inhibition mechanisms of triterpene glycoside of *H. atra* toward orotidine 5-monophosphate decarboxylase. The molecular docking performed that triterpene glycoside of *H. atra* showed active sites with the targeted protein and might blocked the mechanism of uridine synthesis. In silico and in vitro studies of antiplasmodial activity were also reported in a previous study. A derivative compound, N-acylhydrazone, AH5 reported high antiplasmodial activity and less binding energy against some *Plasmodium* proteins, which were 1NHW, 1O5X, 1QNG, PFATP6, 4QOX, 2PML, 4N0Z, 3K7Y, 4B1B, 3T64, 4C81, and 4P7S. The AH5 has a benzene ring in the R-substituent of the compound and might cause less binding energy and high inhibitory activity of plasmodial targeted protein [41]. A recent study reported that 10,977 molecules were selected based on the pharmacophore models and performed inhibitory activity against the PfENR protein [42]. Nine hybrid compounds of 7-substituted 4-aminoquinoline and cinnamic acid were also reported as a novel derivative compounds to inhibit the Pf3D7 chloroquine-sensitive strain. Inhibitory activity ranged from 1.8 to 16 *μ*M, with compound C11 that was the most potential antiplasmodial activity. In silico investigation using frontier molecular orbitals revealed that cinnamic acid favored the LUMO distribution, and the quinoline posed favored HOMO energy. The nine hybrid compounds were also reported to have good pharmacokinetics and drug-like properties [43]; the docking score decreases with compound structural complexity [44]. Those studies summarized that the structures affected the potential activity of inhibiting targeting protein in silico investigation. In comparison, this study also depicted that the nine terpenoid compounds based on the structure-activity relationship (SAR) performed varied antiplasmodial bioactivity, low cytotoxic, mutagenicity, and carcinogenicity potential, and not proper drug-like properties. Interestingly, based on the docking and dynamic simulations, nine triterpenoid compounds have a good inhibitory against PfOMPDC.

Takashima et al. found that fourteen of 156 purchased compounds were identified as inhibitor candidates for PfOMPDC protein with a hit rate that was 9% [39, 40, 41]. A previous study reported the structure of PfOMPDC; the solvent was exposed at the active sites between the phosphodianion and pyrimidine gripper of ScOMPDC. The phosphodianion was exposed at the residue Pro202–Val220, while the pyrimidine gripper of PfOMPDC was mapped at Ala151–Thr165. Interestingly, the active sites of pyrimidine were dominantly identified on the active sites of triterpene glycoside [42, 43]. Previous studies also reviewed and investigated that several natural products showed effectiveness as targeted for parasites. Butyraxanthone B, ancistrolikokine A, ochrolifuanine A, chrobisiamone A, ailanthinone, korupensamine A, 5-prenylbutein, methyl 6-hydroxy-angolensate, calothwaitesixanthone, 7-deacetylkhivorin, and aulacocarpin AP were found targetting *P. falciparum* dihydrofolate reductase [45].

## 5. Conclusion

In conclusion, the triterpene glycoside of *H. atra* is potentially a drug candidate for malaria therapeutic agents. For further investigation, in vitro and in vivo studies were required.

## Figures and Tables

**Table 1:** Compounds, compound identity, LD50, toxicity, and pharmacokinetic prediction.

Compounds	CID	Predicted LD50 (mg/kg)	Toxicity class	GI absorption	BBB permeant	P-gp substrate	CYP1A2 inhibitor	CYP2D6 inhibitor	CYP3A4 inhibitor	LogKp skin permeation (cm/s)
17-Hydroxyfuscocineroside B	25099007	800	4	Low	No	Yes	No	No	No	-14.69
Holothurin A3	71728339	800	4	Low	No	Yes	No	No	No	-15.79
Holothurin A1	102057279	3220	5	Low	No	Yes	No	No	No	-14.65
Calcigeroside B	163105984	4000	5	Low	No	Yes	No	No	No	-14.70
Fuscocineroside C	44559164	3220	5	Low	No	Yes	No	No	No	-14.33
Holothurin B	23674754	3220	5	Low	No	Yes	No	No	No	-11.37
Echinoside A	156831	4000	5	Low	No	Yes	No	No	No	-14.13
24-Dehydroechinoside B	101610324	4000	5	Low	No	Yes	No	No	No	-10.35
Echinoside B	73999936	4000	5	Low	No	Yes	No	No	Yes	-10.17

**Table 2:** Druglikeness prediction and binding energy of triterpene glycoside compounds of *H. atra*.

Compounds	Lipinski	Ghose	Veber	Egan	Muegge	Bioavailability score	Binding energy (kJ/mol)
17-Hydroxyfuscocineroside B	No; 3 violations: MW > 500, NorO > 10, NHorOH > 5	No; 4 violations: MW > 480, WLOGP < −0.4, MR > 130, #atoms > 70	No; 2 violations: Rotors > 10, TPSA > 140	No; 1 violation: TPSA > 131.6	No; 6 violations: MW > 600, TPSA > 150, #rings > 7, Rotors > 15, H‐acc > 10, H‐don > 5	0.17	-1,098.13
Holothurin A3	No; 3 violations: MW > 500, NorO > 10, NHorOH > 5	No; 4 violations: MW > 480, WLOGP < −0.4, MR > 130, #atoms > 70	No; 2 violations: Rotors > 10, TPSA > 140	No; 1 violation: TPSA > 131.6	No; 7 violations: MW > 600, XLOGP3 < −2, TPSA > 150, #rings > 7, Rotors > 15, H‐acc > 10, H‐don > 5	0.17	-1,028.04
Holothurin A1	No; 3 violations: MW > 500, NorO > 10, NHorOH > 5	No; 4 violations: MW > 480, WLOGP < −0.4, MR > 130, #atoms > 70	No; 2 violations: Rotors > 10, TPSA > 140	No; 1 violation: TPSA > 131.6	No; 6 violations: MW > 600, TPSA > 150, #rings > 7, Rotors > 15, H‐acc > 10, H‐don > 5	0.17	-976.3
Calcigeroside B	No; 3 violations: MW > 500, NorO > 10, NHorOH > 5	No; 4 violations: MW > 480, WLOGP < −0.4, MR > 130, #atoms > 70	No; 2 violations: Rotors > 10, TPSA > 140	No; 1 violation: TPSA > 131.6	No; 5 violations: MW > 600, TPSA > 150, #rings > 7, H‐acc > 10, H‐don > 5	0.11	-968.27
Fuscocineroside C	No; 3 violations: MW > 500, NorO > 10, NHorOH > 5	No; 4 violations: MW > 480, WLOGP < −0.4, MR > 130, #atoms > 70	No; 2 violations: Rotors > 10, TPSA > 140	No; 1 violation: TPSA > 131.6	No; 5 violations: MW > 600, TPSA > 150, #rings > 7, H‐acc > 10, H‐don > 5	0.17	-1,002.34
Holothurin B	No; 3 violations: MW > 500, NorO > 10, NHorOH > 5	No; 3 violations: MW > 480, MR > 130, #atoms > 70	No; 1 violation: TPSA > 140	No; 1 violation: TPSA > 131.6	No; 5 violations: MW > 600, TPSA > 150, #rings > 7, H‐acc > 10, H‐don > 5	0.17	-601.18
Echinoside A	No; 3 violations: MW > 500, NorO > 10, NHorOH > 5	No; 3 violations: MW > 480, MR > 130, #atoms > 70	No; 2 violations: Rotors > 10, TPSA > 140	No; 1 violation: TPSA > 131.6	No; 6 violations: MW > 600, TPSA > 150, #rings > 7, Rotors > 15, H‐acc > 10, H‐don > 5	0.17	-1,010.74
24-Dehydroechinoside B	No; 3 violations: MW > 500, NorO > 10, NHorOH > 5	No; 3 violations: MW > 480, MR > 130, #atoms > 70	No; 1 violation: TPSA > 140	No; 1 violation: TPSA > 131.6	No; 4 violations: MW > 600, TPSA > 150, H‐acc > 10, H‐don > 5	0.17	-515.6
Echinoside B	No; 3 violations: MW > 500, NorO > 10, NHorOH > 5	No; 3 violations: MW > 480, MR > 130, #atoms > 70	No; 1 violation: TPSA > 140	No; 1 violation: TPSA > 131.6	No; 4 violations: MW > 600, TPSA > 150, H‐acc > 10, H − don > 5	0.17	-571.91

**Table 3:** Compounds, hydrogen bonds, hydrophobic interaction, unfavorable, and electrostatic interaction of ligand–protein complexes.

Compounds	Hydrogen bond	Hydrophobic	Unfavorable	Electrostatic
Holothurin A1	PRO110 (3.03); TYR111 (3.04); TYR156 (2.07); TYR111 (2.0); TYR156 (2.0); GLU77 (2.7; 2.9; 3.0);	LYS151 (4.5); ILE29 (4.9); PHE79 (3.85)	GLU30 (1.8; 1.5; 1.6); SER76 (1.7; 1.9; 0.7; 1.5); SER113 (2.6)	
Holothurin A3	ARG150 (2.6; 2.3; 2.38; 2.7), GLU30 (2.5; 2.1), ASP179 (2.05), GLU30 (2.18), GLU155 (2.7), GLU181 (1.6), LYS147 (1.75)	ILE29 (4.2), PHE79 (2.88)	GLU155 (2.1; 1.3); GLU26 (1.7); GLU30 (2.6)	
Holothurin B	SER57 (2.57); TYR156 (3.04); SER76 (2.12); GLU26 (1.83)	VAL114 (4.4); PHE79 (5.0; 4.6; 4.07); TYR111 (4.05; 2.24)	ASN144 (2.2; 1.4); LYS147 (2.1; 2.0; 2.6); TYR156 (0.89); GLU30 (1.57; 1.55)	ASP175 (3.06)
Echinoside A		TYR156 (5.4); PHE79 (4.4; 4.5); TYR111 (4.6)	ASN183 (1.7; 1.6; 2.1; 1.9); LYS184 (2.6); GLU26 (1.6; 1.5); PHE79 (1.8; 1.9); TYR111 (2.1; 1.8; 1.6; 1.2; 1.3)	
Echinoside B			GLU26 (2.2; 1.2; 2.0; 1.1); TYR111 (2.0)	ASP61 (3.5)
17-Hydroxyfuscocineroside B	SER76 (2.4); TYR156 (2.2); TYR111 (2.7; 2.0); ASP117 (2.0); SER113 (1.9)	LYS151 (5.0); TYR111 (5.1)	ASP117 (2.1; 1.7; 1.8; 2.04); LYS151 (2.2; 1.7); ASP175 (2.4; 1.9); ILE176 (2.6); SER76 (2.3; 2.2; 2.2; 1.2; 1.2); SER113 (2.1; 1.4; 1.8; 1.4)	ASP175 (4.2); LYS174 (3.0); TYR178 (2.2)
24-Dehydroechinoside B	SER113 (2.5; 2.6); TYR111 (2.7); GLU26 (2.2; 2.7)	LYS27 (5.0)	GLU30 (2.2); TYR111 (1.8; 2.1; 0.8)	GLU77 (3.3)
Fuscocineroside C	LYS27 (2.7); GLU30 (2.6); GLU26 (2.08; 2.1); GLU78 (2.3)	LYS151 (5.3; 4.1)	ASP141 (2.7; 1.6; 2.7); LYS151 (2.2; 1.1; 1.5); GLU26 (2.0; 1.3); TYR111 (2.3; 1.8; 1.8)	ASP141 (3.4), PHE79 (3.82)
Calcigeroside B	LYS27 (2.3); GLU26 (2.4; 1.9;); GLU30 (3.08); GLU155 (2.7)		GLU155 (1.9; 1.99; 1.3; 1.14)	

## Data Availability

The author confirms that there is no supporting data in this study.
